# Near vision examination in presbyopia patients: Do we need good homologated near vision charts?

**DOI:** 10.1186/s40662-016-0061-7

**Published:** 2016-11-10

**Authors:** Wolfgang Radner

**Affiliations:** Austrian Academy of Ophthalmology, Mollgasse 11, A-1180 Vienna, Austria

**Keywords:** Reading charts, Reading test, Reading acuity, Reading speed, Reading performance, Functional vision

## Abstract

Presbyopia correction is mainly concerned with the goal of regaining an uncorrected reading performance. Since historic reading charts do not provide a unique standard that is applicable for the analysis of clinical and scientific reading performance, new standardized reading charts have been developed, in order to provide reading performance analyses analogous to modern single-optotype distance acuity measurements: the Bailey-Lovie Word Reading Chart, the Colenbrander English Continuous Text Near Vision Cards, the MNREAD Charts, and the RADNER Reading Charts. The last three are also meant to measure reading speed, thus allowing detailed analysis of the reading capabilities of the patient’s functional vision. Furthermore, these reading charts can be declared homologated, based on the standards that were published for reading charts by the Visual Function Committee of the International Council of Ophthalmology (ICO) in 1988. Many research studies have shown that by analyzing the reading performance with homologated reading charts, valuable insight into the reading performance of patients suffering from various diseases can be obtained. These reading charts have also been successfully used in presbyopia research. It therefore seems evident that homologated, standardized reading charts facilitate not only research concerning functional vision in many fields of ophthalmology but also international communication about near visual performance. Homologated reading charts are available in almost all languages and have become a valuable tool in analyzing reading performance. We argue in this review that homologated reading charts are clearly a necessity for presbyopia research.

## Backgrounds

Presbyopia is an age-related condition that reduces near visual function through successive losses of accommodation. In addition, distance-corrected pseudophakia results in an iatrogenic presbyopia that results from the implantation of a monofocal intraocular lens (IOL). Since both conditions affect near visual properties, particularly the ability to read, presbyopic and cataract patients share the desire to regain a comfortable reading ability.

Whereas age-related presbyopia usually is corrected with reading glasses, sufficient reading acuity after cataract surgery can be achieved with several types of presbyopic correction: (a) with monofocal IOLs and reading glasses, (b) with contact lenses [1] (c) with multifocal IOLs [2–5] or (d) pseudophakic monovision [6] and (e) with corneal inlays [7]. The accommodative potential of accommodating IOLs, however, seems to be limited [8–10]. Recently, promising results have been obtained with a new accommodative IOL model [11]. A noninvasive pharmacological approach for the treatment of presbyopia is the use of a drug combination in order to improve accommodation with eye drops [12].

Given that reading is an important visual task in our information-based society, and cataract and refractive surgery ideally aims to restore a comfortable reading ability, there is increasing clinical interest in scientifically investigating the postoperative reading performance of such patients [1–23]. It seems evident for such research questions that a reading chart standard, by analogy to distance acuity standards, is required in order to make various reading charts and their various language versions comparable with each other. In other words, reading charts need to be homologated.

Is there a standard that allows us to homologate reading charts and their print sizes? In 1988, the Visual Function Committee of the International Council of Ophthalmology published a standard for reading charts [24] in order to establish homologated reading acuity measures. In short: By analogy to the standards of visual acuity measurements, the print sizes of reading charts have to progress logarithmically. The committee concluded that it is desirable that the test conditions, optotypes, and chart design used are homologated and that the test distance is specified in all instances. They further postulated that for reading charts, continuous text materials are desirable, and they recommended that the typeset material be based upon the distance at which the height of lower-case letters such as “o”, “m”, and “x” subtends 5 min of arc.

Since then, only a few modern reading charts have been built upon these useful standards: (a) the Bailey-Lovie Word Reading Chart [25], (b) the Colenbrander English Continuous Text Near Vision Cards (Precision Vision, Woodstock, IL), (c) the MNREAD Charts [26, 27] (Precision, Vision, Woodstock, IL), and (d) the RADNER Reading Charts [28–30] (NeuMed AG, AT; Precision Vision, Woodstock, IL). The last three reading charts are available in several languages.

An innovative example for homologating a well-recognized reading chart with modern standards is the Oculus Reading Probe, which is a printed chart using long paragraphs. For reading acuity, decimal acuities are given for 25 cm, 32 cm, and 40 cm. The OCULUS Corporation reissued their German reading charts in 2015 and decided to ask the author of this article to collaborate in order to homologate the Oculus print sizes with those of the RADNER Reading Charts that are in accordance with the standards of the ICO committee (the author was responsible for the accuracy of the print sizes). Now, the two leading reading charts in the German-speaking countries provide homologated reading acuity measures. This was a hallmark collaboration in ophthalmology since it was the first time that two different reading chart systems have been homologated so that the print sizes, and therefore the reading acuity measures, have been equalized.

For the present review article, I selected only those reading charts that are in accordance with the standards of the Visual Function Committee of the ICO and with EN-ISO 8596 [25, 31]. From a PubMed search, only those studies were selected that had been performed with such homologated reading charts, and a further selection was made in order to avoid multiple presentations of materials and/or surgical methods. The backgrounds of these homologated logarithmic reading charts are discussed with regard to the advantages they offer for research and clinical purposes in the field of presbyopia.

## Main text

### Historical aspects of reading charts

While clear standards for distance acuity measurements and optotypes were established [32] in the second half of the 19th century, a similar standard has not been developed for reading charts. The historic reading charts that are still used, such as the Jaeger [33], Nieden [34], and Parinaud Charts, suffer from a considerable lack of standardization (Table 1). Their print sizes (letter heights) are not standardized and do not logarithmically progress because of the limitations of earlier printing techniques. Whereas today it is possible to print letter heights with an accuracy of approximately 0.01–0.03 mm [28, 29], the historic charts have often been printed only with the limited print sizes available for hot-lead typesetting. This limitation could be the explanation for the many different versions of the English Jaeger charts [35], which are hardly comparable with each other and are not at all comparable to the versions in German or other languages.

**Table 1:** Reading acuity Equivalents of Modern and Historic Reading Charts

Modern homologated reading charts		Parinaud	Jaeger German	Nieden
logRAD logMAR	Decimal 32 cm	Decimal 32 cm	Decimal 32 cm	Decimal 32 cm
−0.2	1.6	–	–	–
−0.1	1.25	–	–	–
0.0	1.0	–	–	–
0.1	0.8	–	–	–
		P1.5 = 0.72	–	–
0.2	0.63	–	J1 = 0.63	N1 = 0.61
				N2 = 0.59
0.3	0.5	P2 = 0.48		N3 = 0.46
0.4	0.4	P3 = 0.40	J2 = 0.43	N4 = 0.40
			J3 = 0.38	N5 = 0.37
0.5	0.32	P4 = 0.33	–	–
0.6	0.25	P5 = 0.29	J4 = 0.27	N6 = 0.29
			J5 = 0.25^a^	N7 = 0.27
			J6 = 0.25^a^	N8 = 0.25
		P6 = 0.23	J7 = 0.23	
0.7	0.2	–	J8 = 0.20	–
0.8	0.16	P8 = 0.18	J9 = 0.18	N9 = 0.17
		P10 = 0.16		
0.9	0.125	P14 = 0.12		

^a^J5 and J6 have the same print size but differ in font types

The Jaeger charts were originally developed by the Viennese Professor Eduard Jaeger (1818–1884) in 1854 [33]. However, even the original versions do not represent a comparable international standard because the German version was printed with Gothic letters, while an Antiqua font type was used for the English version. In the current version of the German Jaeger charts, there are a number of nonconformities with modern requirements for visual acuity tests (Table 1), including the fact that paragraphs J5 and J6 have the same print size (1.95 mm in height) but different font types. J1 is just comparable to a decimal acuity of 0.63 (Snellen: 20/32) at 32 cm, and J2 corresponds to a visual acuity of 0.43 (Snellen: 20/47) instead of 0.5 (Snellen: 20/40). Between J3 and J4, the print sizes differ by almost 2 log units.

Because historic reading charts like the Jaeger, Parinaud, and Nieden Charts lack useful standards, it seems evident that the evaluation of reading performance using these charts has never been applicable for research purposes. Therefore, historic reading charts should be considered obsolete for the purposes of research and medical documentation.

### Modern logarithmic reading charts

All of these modern reading charts use a logarithmic progression of print sizes and are homologated in accordance with the standards established by the International Council of Ophthalmology [24] and EN-ISO 8596 [31].

#### The RADNER Reading Charts

**Fig. 1:**
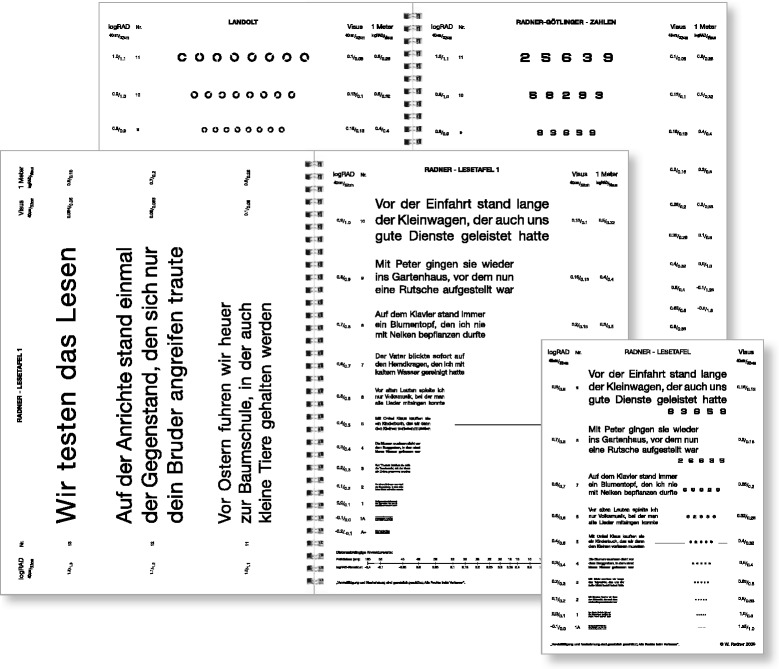
RADNER Reading Charts, as exemplified by the German version (four text reading charts, a page with Landolt rings, and a page with numbers are provided in the booklet). (Original size: big issue, DIN A4 29.7 cm × 21.0 cm; small issue, DIN A5 21.0 × 14.8 cm)

To achieve the best accordance with optotype standardization [24, 31, 36, 37], the RADNER Reading Charts (Fig. 1) make use of the author’s original concept of “sentence optotypes” in order to provide clear definitions for the (a) test items, (b) stop criterion, (c) difficulty, and (d) reading length, and to keep the geometric proportions between the test items as constant as possible [28, 29].

##### The concept of sentence optotypes

The standardization of test items by statistical selection has been introduced for reading charts because the statistical definition of test items is a necessity for a medical test used in patient care [28, 29]. The sentences optotypes of the RADNER Reading Charts are highly comparable in terms of the number of words (14 words), word length, number of syllables, number of characters, position of words, lexical difficulty, and syntactical complexity. The position of words is defined by specified rules [28, 29]: e.g., the first line (5 words) starts with a word of three letters and one syllable, followed by a noun with two syllables in position two or three. The second line also starts with a word of three letters and one syllable, which is followed by a noun of 10 letters and 3 syllables. Then the relative clause starts with 3 short one-syllable words, and so on. Such sentence optotypes of 3 lines (main clause/relative clause) incorporate 82–84 characters including spaces (27–29 characters per line) and 22–24 syllables. By introducing a narrow “reading length interval”, the most equivalent sentence optotypes (*n* = 38) were statistically selected by testing a group of 198 volunteers with respect to reading length and difficulty [27, 28]. The Cronbach’s alpha and the corrected item total correlation were well above statistically required limits [28, 29]. The reading speed correlated well with that obtained for long paragraphs, indicating a good validity of these test items.

##### Standardization of the reading charts

For standardizing the RADNER Reading Charts, a methodical design for reading chart standardization including Bland-Altman analyses was established in 2004 to investigate their test-retest reliability and inter-chart reliability and to evaluate a reading chart through variance component analysis (test-retest interval: 4 weeks) [30]. The results have demonstrated that these reading charts provide highly reproducible measurements of reading acuity and speed in individuals with no, moderate, or increased visual impairment. In addition, they have shown that the reading charts provide reliable, reproducible, and comparable measurements of reading performance for clinical practice and scientific surveys.

The sans serif Helvetica typeface was used for the reading charts. All notations (decimal, Snellen, M-units, and logRAD) are given for 40 cm and 32 cm (in the German version, these are also given for 1 m). Except for logRAD, which is given in all language versions, the notations shown on the charts depend on the tradition of reading acuity determinations of the countries in which the particular language is spoken. A logRAD adjustment scale for different reading distances is provided on every chart (range: 4 cm to 50 cm).

The concept of sentence optotypes has been applied to 11 different languages (a total of 1253 volunteers have been tested in order to standardize the sentence optotypes in the 11 languages). The RADNER Reading Charts are commercially available in German, Spanish, English, French, Dutch, Italian, Swedish, Danish, Portuguese, Turkish and Hungarian, with further languages in progress.

#### The Bailey-Lovie Word Reading Charts

In 1980, Bailey and Lovie published the Bailey-Lovie Word Reading Charts that were designed to determine reading acuity and speed in one simultaneous examination with a reading chart [25]; this principle has also been applied to the MNREAD and the RADNER Reading Charts. Bailey and Lovie designed a word-reading chart with a logarithmic size progression and used unrelated words. Following the recommendations of the British Faculty of Ophthalmologists [38, 39], they used the Times Roman typeface. They further decided to use 4-, 7-, and 10-letter words at each size level, based on the observation that in patients with age-related macular degeneration (AMD), the word length can affect the readability (some patients prefer longer words, others shorter ones). The words and word order were selected with the intention of having the first letters of the words evenly distributed over the whole alphabet. The frequency of word use also became a selection criterion, and care was taken to avoid obvious syntactic associations between adjacent words [40]. On the charts, print sizes were labeled in the N-notation (points) and logMAR values given for 25 cm.

#### The MNREAD Charts

Legge and colleagues [41] were the first to use single sentences for a computer-aided test of reading speed, first called the Minnesota low-vision reading test. Subsequently, a chart version was developed using short sentences over a wide range of print sizes in 1993, called the MNREAD test [19]. This test incorporated the concept of “standard-length word” introduced by Carver [42, 43]. The sentences of the MNREAD tests are characterized by their length, which is defined as 60 characters including spaces and an implied period at the end of a sentence [26]. Based on a study by Carver [42], this length turned out to be convenient for scoring reading errors and speed when a “standard-length word” is defined to have 6 characters. In this case, a 60-character sentence consisted of 10 standard-length words. Using standard-length words helps minimize the differences in scoring that occur as a result of the different word lengths found in different sentences [26, 41–43]. The MNREAD Charts are available in several languages and they give the logMAR notation, Snellen notation, and M-units for 40 cm.

Similar to the test re-test reliability analysis performed for the RADNER Reading Charts [30], a Bland-Altman test re-test analysis (test-retest on the same day) was also performed by Subramanian et al. in 2009 [44] for the two MNREAD Charts. Virgili published the coefficient of repeatability obtained for a group of children with the two Italian MNREAD Charts. The studies showed good repeatability in visually impaired adults and children [45].

#### The Colenbrander English Continuous Text Near Vision Cards (Fig. 2)

**Fig. 2:**
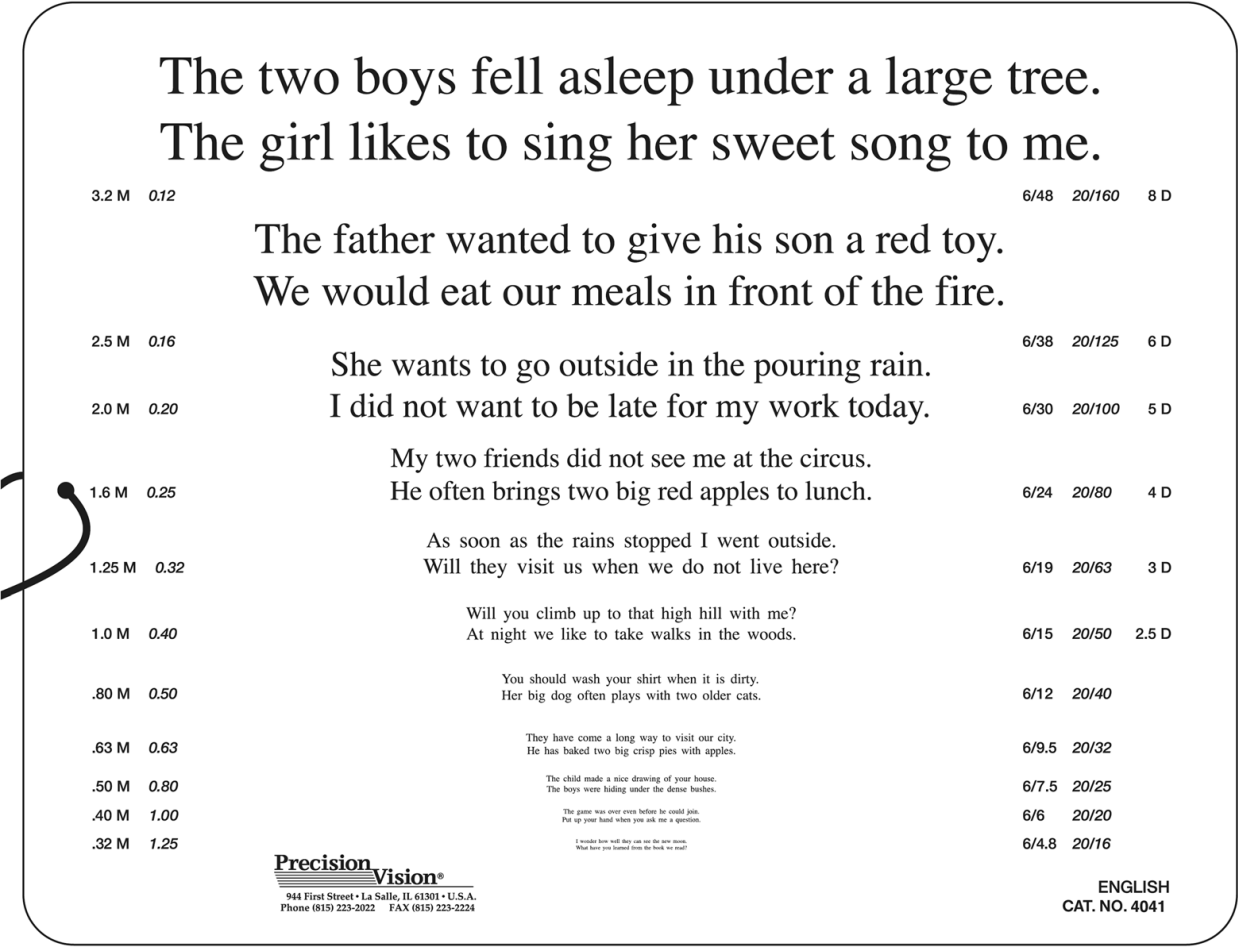
The Colenbrander English Continuous Text Near Vision Cards (original size: 23.0 cm × 18.0 cm). Printed with the permission of August Colenbrander

The Colenbrander English Continuous Text Near Vision Cards (Precision Vision, Woodstock, IL) are also logarithmically scaled; they are available in 11 languages. For use at 40 cm, they cover decimal acuities from 0.063 to 1.25 and also give the Snellen notation and M-units. A logMAR notation is not given. To maintain the correct reading distance, a 40-cm cord is mounted on the cards, and for use in low vision, they come with a ruler to facilitate use at shorter distances for lower acuity levels. The test sentences have 44 characters including spaces and 9 to 11 words. From decimal acuities from 0.063 to 0.1, one sentence is presented per print size, and for 0.12 and smaller, two sentences are presented. These reading cards are also available as mixed-contrast cards; high and low contrast (20 % Weber) are presented side-by-side on the same card.

### Reading parameters

Standardized logarithmic reading charts give better insights into the visual performance of our patients than do conventional or historic charts. In addition to reading acuity, the reading acuity score, maximum reading speed, mean reading speed, and several other reading parameters can be analyzed. An interesting parameter is the “critical print size” (CPS), which is defined either as the smallest print size read with normal reading speed [2, 28–30] or in such a way that all smaller print sizes of the chart are read at a speed below the average reading speed of the largest preceding sentences minus 1.96 times the standard deviation [44]. However, in the variant component analysis performed by Stifter et al. for the CPS [30], the patients investigated accounted for only 31 to 54 % of the entire variance. The higher this percentage, the more likely it is that the test is dependent on the person’s reading ability, as is shown for reading acuity: 85 to 94 %. In comparison to the other variables, the variance component analyses revealed that, for the CPS, a considerable proportion of the variability came from unidentified sources. One explanation for this difference might be that the CPS is not a measurement like reading acuity or speed since it has to be set by the examiner at the smallest print size the patient can read with optimal reading speed. For the statistical definition of the CPS [44], it was also found that the coefficient of repeatability was considerably weaker than that of reading acuity and reading speed.

**Fig. 3:**
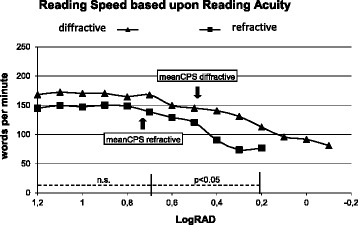
Mean reading speed, based on reading acuity and mean critical print size of two different multifocal IOLs (MIOL). Forty eyes per group were investigated. Note the significant difference in the mean reading speed between the diffractive and the refractive MIOL at reading acuities ranging from logRAD 0.7 to 0.3 (n.s., not significant)

“Reading speed based upon reading acuity” (Fig. 3) and the “logMAR/logRAD ratio” can also provide useful information about functional vision, as can the reading score [10], which was developed to compare the reading speed obtained under different reading conditions.

### Stop criteria

A further advantage of the standardized reading charts is that they permit the introduction of a stop criterion, which can be freely chosen with regard to the requirements of a particular study design. For the RADNER Reading Charts, a stop criterion of 20 s is recommended [28], which represents a reading speed of about 40 wpm, the higher limit of spot-reading; whereas the limit for fluent, sense-capturing reading is 80 wpm. A speed of 80 wpm seems to be too short for a stop criterion, since it represents a reading time of just about 7 s per sentence for the MNREAD Charts and 10 s per sentence for the RADNER Reading Charts. In normal-sighted persons, these speeds per sentence represent the reading speed at a print size that is close to the CPS; with this limit, the patient’s full visual potential (i.e., best reading acuity) cannot be shown. However, the best possible reading acuity is an important result, as is the best distance acuity. It is also a valuable indicator of a comfortable reading performance. Reading acuity should therefore be determined in detail, in accordance with the procedures used for single-optotype distance acuity [24, 31].

### LogRAD, a modern reading acuity measure

Since from a psychophysical point of view reading acuity involves a different visual task than single-optotype distance acuity, the author of this article suggests the use of different definitions for different tasks and has introduced the term “logRAD” (log-Reading Acuity Determination) for reading acuity measures, which is the reading equivalent of logMAR [28, 29].

This concept was found to be convenient because it avoids the confusion between distance and reading acuity that occurs when logMAR is used for both distance and reading acuity. In addition, this differentiation of distance and reading acuity follows the principle that different definitions should be used for different functional properties, as is the case for physics terms used in everyday life (e.g., Hz, Watt, kg, kp, meter, seconds).

Several notations are in use for measuring reading acuity: the (a) decimal notation, (b) Snellen notation, (c) M-notation, (d) N notation, (e) logRAD notation and (f) logMAR notation. Except for the N-notation, all of these notations are based on the same definition of the visual angle that was introduced by Snellen in 1862 [36], and all of them are calculated based on the mathematical relation between the visual angle and the testing distance. Furthermore, all of these notations are related to the “minimal angle of resolution” of the eye. Reading acuity, however, is a visual task that is different from that of single-optotype distance recognition, and the definitions of the print sizes are based on the size of lower-case letters. Therefore, it seems to be useful to use different terms for distance acuity (logMAR) and reading acuity (logRAD).

### Clinical aspects of homologated reading charts

The first study in which the reading performance with multifocal IOLs was investigated with a standardized reading chart was performed with the RADNER Reading Charts [2]. Since then, a number of studies have been performed with these standardized logarithmic reading charts and have shown that it is possible to obtain detailed information about the reading performance achieved with bi-, multi-focal, and accommodative IOLs [2–5, 9–11, 16, 17, 22], corneal inlays, [7], monofocal IOLs [18, 23], and following LASIK/LASEK [15] or refractive laser treatment for presbyopia [18–20]. Interesting insights into the reading performance of cataract patients have also been obtained with the Bailey-Lovie Word Reading Charts [14] and with the MNREAD Charts as e.g. for two accommodating IOLs [16] and for reading performance of diffractive MIOLs obtained from patients of working age [22].

In addition, the RADNER Reading Charts have also been used to investigate the reading performance of patients suffering from many diseases, including AMD [46–48], amblyopia [49, 50], infantile nystagmus [51], uveitis [52], and telangiectasia type 2 [53], as well as that of patients who have undergone various surgical treatments [54–59].

Many clinical research studies have used standardized and homologated reading chart systems, indicating that they have become a useful tool for investigating reading performance. Such results not only allow the comparison of different conditions within a particular study, but they also allow the comparison of the results of studies and clinical results. Also, readers of the articles have a clear idea of the print sizes behind homologated reading acuity measures.

## Conclusions

Bailey and Lovie-Kitchin concluded that “reading of words or sentences is clearly a more complex function than is reading the widely spaced letters of a distance acuity chart” [25]. They further summarized that, as “compared to isolated letters, the individual letters within words are more difficult to recognize because of interactions with closely packed neighboring letters” [60, 61]; the more important element in reading was found by Bouma to be the recognition of letter and word sequences [62–64].

It therefore is not surprising that routine single-optotype distance visual acuity tests have been shown to be poor predictors of reading performance and, thus, cannot elucidate the full functional impairment of several ophthalmic diseases [65, 66].

In presbyopia research, modern standardized reading charts allowed the investigation of reading performance in a standardized manner. In particular, reading speed evaluation based upon reading acuity, the critical print size, and the mean and maximum reading speeds has provided interesting insights into the near-visual performance of presbyopic patients prior to and following therapy. Clinical studies have investigated the reading performance of patients with various models of multifocal and accommodating IOLs [2–5, 9–11, 16, 17, 22], corneal inlays [7], or following laser refractive surgery [19–21]. Also, the reading performance of cataract patients has been analyzed in detail [17, 67, 23], and reading tests have been shown to be useful for estimating potential acuity [14, 68]. Another study has investigated the reading performance of cataract patients who had received a monofocal IOL, with or without glasses, under bright and dim light conditions [23].

In summary, it is quite evident that homologated, standardized reading charts such as the Bailey Lovie Reading Word Reading Charts [25], the Colenbrander Cards, the MNREAD Charts [26, 27], and the RADNER Reading Charts [28–30] facilitate not only research concerning functional vision in many fields of ophthalmology but also international communication about near-visual performance. Homologated reading charts are available in almost all languages and have become a valuable tool for analyzing reading performance.
